# QuickStats

**Published:** 2013-09-06

**Authors:** FROM THE NATIONAL CENTER FOR HEALTH STATISTICS

**Figure:**
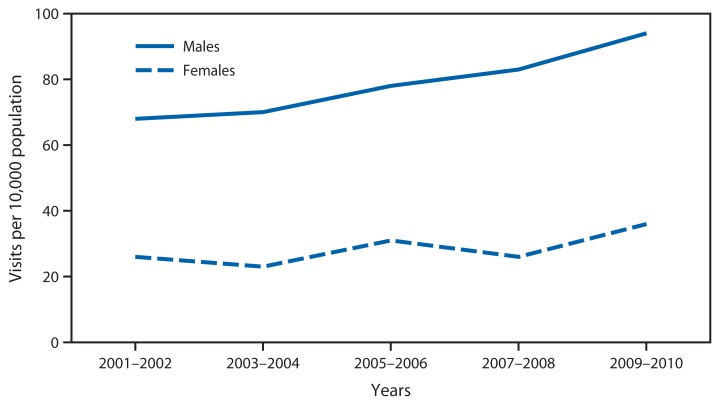
Rate^*^ of Emergency Department Visits for Alcohol-Related Diagnoses,^†^ by Sex — National Hospital Ambulatory Medical Care Survey, United States, 2001–2002 to 2009–2010 ^*^ Rate per 10,000 population, based on 2-year annual average. Rates were calculated using U.S. Census Bureau 2000-based postcensal noninstitutionalized civilian population estimates. ^†^Defined as any-listed diagnosis codes 291, 303, 305.0, 357.5, 425.5, 535.3, 571.0–571.3, and 790.3, and any-listed cause of injury code E860.0 based on the *International Classification of Diseases, Ninth Edition, Clinical Modification*. Not included are emergency department visits that might be attributed to alcohol use, such as falls, motor vehicle crashes, and other types of injuries/conditions.

From 2001–2002 to 2009–2010, the rate of emergency department visits for alcohol-related diagnoses for males increased 38%, from 68 to 94 visits per 10,000 population. Over the same period, the visit rate for females also increased 38%, from 26 to 36 visits per 10,000 population. Throughout the period, the visit rate for males was higher than the visit rate for females.

**Source:** CDC. National Hospital Ambulatory Medical Care Survey. Available at http://www.cdc.gov/nchs/ahcd/ahcd_questionnaires.htm.

**Reported by:** Anjali Talwalkar, MD, atalwalkar@cdc.gov; Farida Ahmad, MPH.

